# DONALD P. KENT AND ROBERT W. KLEEMEIER AWARD LECTURES

**DOI:** 10.1093/geroni/igac059.1247

**Published:** 2022-12-20

**Authors:** Peter Lichtenberg

## Abstract

The Donald P. Kent Award lecture will feature an address by the 2021 Kent Award recipient Luigi Ferrucci, MD, PhD, FGSA, of the National Institute on Aging. The Kent Award is given annually to a member of The Gerontological Society of America who best exemplifies the highest standards of professional leadership in gerontology through teaching, service, and interpretation of gerontology to the larger society. The Robert W. Kleemeier Award lecture will feature an address by the 2021 Kleemeier Award recipient Kenneth F. Ferraro, PhD, FGSA, of Purdue University. The Kleemeier Award is given annually to a member of The Gerontological Society of America in recognition for outstanding research in the field of gerontology.

